# The “gift effect” on functional brain connectivity. Inter-brain synchronization when prosocial behavior is in action

**DOI:** 10.1038/s41598-020-62421-0

**Published:** 2020-03-25

**Authors:** Michela Balconi, Giulia Fronda

**Affiliations:** 1 Research Unit in Affective and Social Neuroscience, Catholic University of the Sacred Heart, Milan, Italy; 2 Department of Psychology, Catholic University of the Sacred Heart, Milan, Italy

**Keywords:** Cognitive neuroscience, Social behaviour

## Abstract

The gift exchange represents a moment that characterizes interpersonal interactions. In particular, research in psychological and neuroscientific fields aimed to observe the social function of gift exchange. Specifically, the present study aimed to investigate the effects of prosocial behavior, experienced during gift exchange, on individuals’ cognitive performance and brain activity. To this aim, behavioral performance and neural activity of 15 dyads of participants, with a consolidated friendship, were collected during the execution of an attentional cooperative task before or after a gift exchange. Individuals’ brain activity was recorded through the use of Functional Near Infrared Spectroscopy (fNIRS) in hyperscanning. Results showed an increase of perceived cooperation and cognitive performance, in terms of accuracy (ACC), after gift exchange. The increase of interpersonal tuning and cooperation was also shown by neural activity with an increase of oxygenated hemoglobin (O2Hb) intra-brain and inter-brain connectivity in the dorsolateral prefrontal cortex (DLPFC) following the gift exchange. Moreover, from ConIndex analysis emerged an increase of inter-brain connectivity compared to intra-brain in DLPFC area. The present study, therefore, highlights how prosocial behavior can have positive effects on cognitive performance improvement and interpersonal relationships and neural coordination strengthen, increasing intra and inter-brain connectivity mechanisms.

## Introduction

The social exchange represents a significant moment of interaction that characterizes individuals’ lives. Due to the centrality of exchange moments in everyday life, several anthropological studies have investigated exchange relationships^1^. In addition, research in sociological and psychological fields has identified exchange moments as characterizing interpersonal interactions^2–5^. In particular, the exchange presents itself in various forms, including the giving of materialistic objects as gifts, which builds and strengthens interpersonal relations and social ties^6,7^. Specifically, the exchanging of gifts, which are defined as goods or services voluntarily provided to another person or group^7,8^, has long been considered an activity that strengthens individuals’ social interactions^9^ and cooperative bonds^10^. In this sense, the gift assumes a social function^3^, constituting experiences connoted by a plurality of emotions^8,11,12^ such as joy and gratitude, which facilitate the construction of reciprocal relationships^13–15^ and the implementation of prosocial behaviors both in donors and receivers^16–19^.

Indeed, a selfless gift is considered as representative of a specific prosocial behavior, able to strength individuals’ sense of reciprocity and cooperative ties^20–25^, increases inter-agents’ behavioral coordination through the implementation of specific neurophysiological modulation, such as brain-to-brain coupling mechanisms that occur when both individuals experience the same moods and perceptions^26–30^. As demonstrated by previous studies, joint action and prosocial conditions development increases inter-agents’ inter-cerebral synchronization^31^, improving individuals’ behavioral and cognitive efficiency^29,30,32–37^. In particular, this increase of inter-cerebral synchronization occurs in specific cerebral areas, such as frontal regions that are the most implicated in social, prosocial and cooperative mechanisms^38–40^. Specifically, a portion of prefrontal cortex, the DLPFC, appears to be particularly implicated in interpersonal exchange mechanisms^41,42^ in action planning and cooperation^43–45^.

In light of this evidence, in order to investigate the influence of neural synchronization mechanisms on cognitive performance and cooperation levels after a gift exchange, we have implemented an experimental paradigm in which coupled participants were asked to exchange a gift during the performance of a cognitive task under explicit cooperation conditions. Coupled individuals’ neural responses were recorded through the use of fNIRS in hyperscanning.

Specifically, fNIRS consists of a neuroimaging technique that, thanks to its usability and portability, allows for the investigation of brain activity in more realistic and ecological interaction conditions^46,47^. Indeed, as shown by different studies, fNIRS has contributed to the investigation of the neural mechanisms underlying various social relationship processes^48–50^.

Moreover, the use of hyperscanning technique has allowed us to abandon the classic individual investigation approach and to embrace the use of a “two-person neuroscience.” In particular, hyperscanning consists of a recent paradigm that has demonstrated its effectiveness in cognitive and social studies^34,35,37,50^, allowing the recording of two individuals’ simultaneous brain activity during the performance of a shared task or the development of an interaction^50,51^, providing information about individuals’ inter-brain functional connectivity.

The latter can be defined as the correlation between two-time series^52^ that reflects agents’ neuronal activations^32,53^ and provides information on spatially distant neurophysiological events^54^.

In particular, functional connectivity gives information about the synchronic and diachronic aspects, measured by cross-correlations or coherence, underlying individuals’ interaction^33^ related to an increase of intra-brain and inter-brain connections that allow the creation of implicit coupling mechanisms^39,42,55^ and interpersonal coupling dynamics^47^. The advantages of using hyperscanning technique to obtain information about inter-brain connectivity, interpersonal coupling and social understanding processes^32^ have been demonstrated by different studies that have investigated individuals’ cerebral synchronization underlying different joint actions moments such as cooperation^32,37^ and empathic and prosocial behaviors^30,56^.

The present study, therefore, aimed to investigate whether and how individuals’ cognitive performance and brain activity improve following the implementation of prosocial behavior characterized by a gift exchange. In particular, we aimed to explore whether the specific moment of gift exchange (at the beginning or in the middle of the interaction) could influence the subsequent responses of both individuals involved in the interaction. To this end, two gift exchange moments were hypothesized: at the beginning of the task and in the middle of the task.

In particular, we theorised that an early gift exchange could immediately influence the relationship with positive effects on behavioral performance compared to a subsequent gift exchange. This effect would be present when gift exchange was performed, independently from the time (early or late) the gift was administrated. Therefore we expected to observe an increase in dyadic tuning and cooperation self-perception following the gift exchange due to the fact that this moment involved experiencing positive emotions, which increases the implementation of prosocial behaviors^16,18,19,57^, reinforcing the sense of reciprocity between individuals^20–22,24,25,58^. However, due to the increasing effect of synchronization and the development of a higher cooperation during the task, we expected an ampler effect in response to early gift exchange compared to late gift exchange. This sort of long-lasting effect was expected for behavioral measures and connectivity measures.

In addition, concerning behavioral responses, we expected to observe an increase in accuracy following the gift exchange. Indeed, as shown by previous studies, the increase in cooperation, coordination and synchronization provided by the implementation of prosocial behavior improves behavioral and cognitive efficiency^31–33,59–61^.

Regarding neural responses and brain connectivity, we expected to observe, following gift exchange, an increase of O2Hb intra-brain and inter-brain connectivity in frontal brain areas and in particular in the DLPFC region that appears to be involved in interpersonal exchange mechanisms^41,42,62^, action planning processes and cooperation^38,44,45,63^. As demonstrated by previous studies, indeed, during joint actions, an increase of intra-brain neural synchronization and inter-cerebral synchronization occurs between individuals involved in the exchange, also leading to a better behavioral coordination^27^. Therefore we expected a significant increased performance after gift exchange and a concomitant higher synchronization for both intra- and inter-brain connectivity measures.

## Methods

### Participants

The research was conducted on 15 dyads of female participants, for a total of 30 university students (Mage = 22.31; SDage = 1.66). The participants coupled in dyads had a consolidated friendship, who attended and saw each other regularly.

Participants were recruited through the use of the following inclusion criteria: consolidated friendship and absence of psychiatric or neurological diseases. On the contrary, participants with cognitive deficits, a clinically relevant stress level and stressful events occurrence during the last 6 months were excluded from the research. All participants took part in the study after signing the written informed consent. For the research conduction, the principles and guidelines of Helsinki Declaration were respected and also the approval was obtained from the local ethics committee of the Department of Psychology of the Catholic University of Milan.

### Procedure

**Figure 1:**
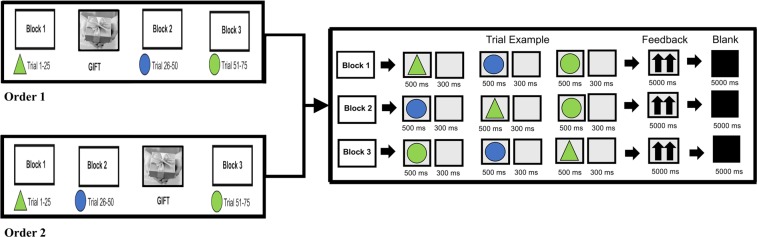
The figure shows the task experimental procedure for order 1 and 2 in which change the moment of gift exchange during the execution of the three blocks consisting in a selective attentional task.

To carry out the research participants were required to seat side by side in front of a computer located 60 cm away from them that are separated by a black divider to prevent the possibility to communicate, looking at each other or influencing each other during the task performance. The coupled participants were invited to carry out a joint activity, consisting in a computerized task performance before (at the activity beginning) or after (in the activity middle). For the gift exchange, a member of the dyad was identified as donor, who was asked to previously choose a gift to consign to her partner. The other member of the dyad, instead, was designated as receiver that receive the gift given from the donor. The gift delivery was randomized among the participants dyads, making sure that for some couples the gift exchange took place before the beginning of the task; while, for the remaining in the middle of the task. This involved the development of two experimental procedures depending on the moment of gift exchange. Specifically, the first procedure (order 1) was structured as follows: the execution of block 1 (a basic condition), the gift exchange and finally the execution of blocks 2 and block 3. On the contrary, the second procedure (order 2) required participants the execution of blocks 1 and 2, the gift exchange and the execution of block 3 (Fig. 1).

In particular, blocks 1, 2 and 3 required participants to perform a task consisting of a selective attention game to be played in cooperation. The task represented a modified version of a previous validated version of the task^33,39,64,65^, in which we have opted for a cooperative condition with a specific moment of gifts exchange. In particular, the task was administered through the use of the E-Prime 2.0 software (E-prime2 software, Psychology Software Tools Inc., Sharpsburg, PA, USA), and required participants to memorize a target stimulus (green or blue triangle or circle) which was to be subsequently recognized among other stimuli, by pressing the left/right keys on the computer keyboard. The stimuli were presented on the screen for 500 msec with an inter-stimulation interval (ISI) of 300 msec and an inter-trial interval (ITI) lasting 5000 msec. The aim of the task, specifically, required participants’ dyad to synchronize their responses in terms of speed response and ACC. To this aim, after the appearance of three stimuli, participants’ dyads received a feedback concerning their level of good cooperation pictured by two up arrows.

Participants’ perception of cooperation during the execution of the three blocks was investigated through the administration of a questionnaire before and after the gift exchange to observed possible differences in the perceived cooperation degree. In particular the questionnaire contained the following items: “What was the perception of your workmate in the first phase of the game?”, “What was the perception of your workmate in the second phase of the game?”, “What was the perception of your cooperation in the first phase of the game?”, “What was the perception of your cooperation in the second phase of the game?”. Individuals responses were codified by three expert judges using a Likert scale.

### fNIRS recording and signal processing

**Figure 2:**
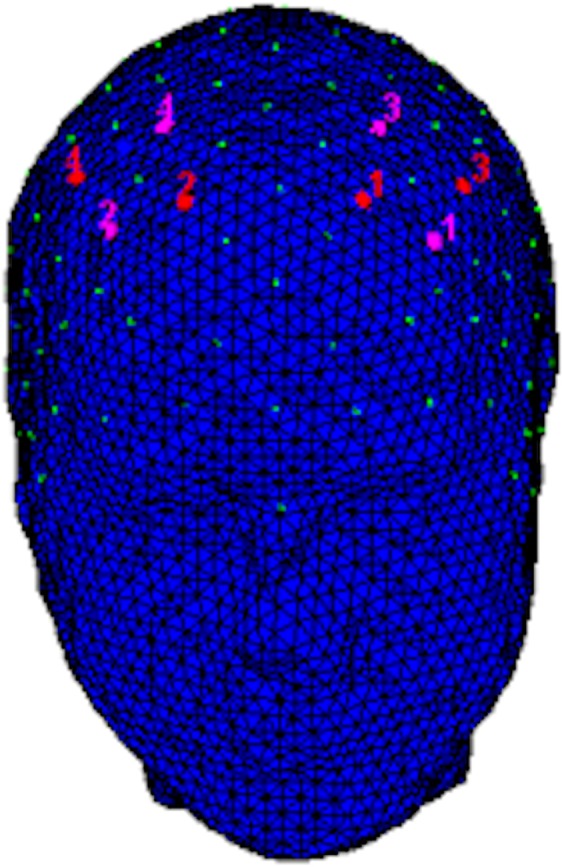
The figure shows the fNIRS montage. The sources (red color) were placed on positions FC3–FC4 and F1–F2, while detectors (pink color) were placed on FC1–FC2 and F3–F4 positions.

For the recording of hemodynamic responses, a NIRScout system was used (NIRx Medical Technologies, LLC, Los Angeles, California) with an 8-optode matrix (4 sources and 4 detectors) that was placed on the frontal and prefrontal regions of each individual according to the international 10/5 system using a fNIRS cap. Specifically, for the signal recording optodes were placed with a distance of 30 mm as follows: sources in F1-F2 and FC3-FC4 positions, detectors in F3-F4 and FC1-FC2 positions (Fig. 2). Moreover, a near-infrared light at 760 and 850 nm was used.

The optodes montage allowed to record the following channels: Ch1 (FC3-F3), Ch2 (FC3-FC1), Ch3 (FC4-F4), Ch4 (FC4-FC2), Ch5 (F1-F3), Ch6 (F1-FC1), Ch 7 (F2-F4), Ch8 (F2-FC2). ROI were calculated for both left/right homologous sides: the values obtained from Ch1 and Ch3 correspond to the left and right DLPFC (Broadman Area 9); the values obtained from Ch2 and Ch4 correspond to the left and right Dorsal Pre-Motor Cortex (DPMC, Broadman Area 6); the values obtained from Ch5 and Ch7 correspond to the left and right Frontal Eye Fields (FEF, Broadman Area 8); the values obtained from Ch6 and Ch8 correspond to the left and right Superior Frontal Gyrus (SFG, Broadman Area 6)^66^. After the acquisition of a 120 seconds baseline, the concentration of oxygenated (O2Hb) and deoxygenated (HHb) hemoglobin were continuously recorded with a sampling frequency of 6.25 Hz. For the signal analysis nirsLAB software (v2014.05, NIRx Medical Technologies LLC, 15Cherry Lane, Glen Head, NY, USA) was used. The O2Hb and HHb raw data for each channel were digitally filtered to a filtered band at 0.01–0.3 Hz.

### Statistical analysis

Two analyses’ orders were performed to behavioral (Dyadic tuning; Perceived cooperation; ACC; RTs) and neurophysiological (fNIRS: O2Hb, HHb measures) dependent measures. For all the ANOVA tests, for the freedom degree correction, Greenhouse–Geisser epsilon was used. Post-hoc comparisons (contrast analyses) were applied to the data. A Bonferroni test was applied for multiple comparisons. In addition, the normality of the data distribution was preliminary tested (kurtosis and asymmetry tests). The normality assumption of the distribution was supported by these preliminary tests.

### Behavioral performance measures

For each subject, concerning the execution the task, ACC and RTs were obtained through the use of the E-prime Software. Specifically, for the calculation of ACC, the percentage of correct responses on the total responses were considered, while RTs were considered starting from stimulus presentation.

Then, two mixed-model ANOVAs were applied to ACC and RTs with Blocks (1 vs 2 vs 3) as repeated factor, Condition (Cond: order 1 vs order 2) and Role (Role: donor vs. receiver) as between factors.

### fNIRS analyses

For each channel, the mean concentration of O2Hb and HHb was calculated by averaging data across the three blocks. The effect size in every block was calculated for each channel and subject as the difference of the means of the block (m2) and the baseline (m1) divided by the standard deviation (sd) of the baseline: d = (m2-m1)/sd (Cohen’s d value), according to the mean concentrations in the time series^43^. The procedure was applied to both O2Hb and HHb concentration variations. Then, two mixed-model ANOVAs were applied to O2Hb and HHb measures with Condition (Cond: order 1 vs order 2) as between factor, Blocks (1 vs 2 vs 3) and ROI (4) as repeated factors.

### Functional connectivity analysis

The second statistical set of analyses (intra-brain and inter-brain connectivity applied to neurophysiological O2Hb, HHb measures), which consisted in four different steps: at first, to obtain intra- and inter-brain connectivity, the partial correlation coefficient Π_*ij*_ was computed to obtain functional connectivity indices. They were obtained by normalizing the inverse of the covariance matrix $\Gamma ={\Sigma }^{-1}$:

$$\Gamma =({\Gamma }_{ij})={\Sigma }^{-1}\,{\rm{i}}{\rm{n}}{\rm{v}}{\rm{e}}{\rm{r}}{\rm{s}}{\rm{e}}\,{\rm{o}}{\rm{f}}\,{\rm{t}}{\rm{h}}{\rm{e}}\,{\rm{c}}{\rm{o}}{\rm{v}}{\rm{a}}{\rm{r}}{\rm{i}}{\rm{a}}{\rm{n}}{\rm{c}}{\rm{e}}\,{\rm{m}}{\rm{a}}{\rm{t}}{\rm{r}}{\rm{i}}{\rm{x}}\,{\rm{p}}{\rm{a}}{\rm{r}}{\rm{t}}{\rm{i}}{\rm{a}}{\rm{l}}\,{\rm{c}}{\rm{o}}{\rm{r}}{\rm{r}}{\rm{e}}{\rm{l}}{\rm{a}}{\rm{t}}{\rm{i}}{\rm{o}}{\rm{n}}\,{\rm{m}}{\rm{a}}{\rm{t}}{\rm{r}}{\rm{i}}{\rm{x}}$$

It quantifies the relationship between two signals (i, j) independently from the other^67^. More specifically, these measures represent the covariance of two signals, that allows to calculate the partial correlation coefficients between two series of data, in response to specific conditions (the experimental conditions).

Then, ANOVAs were applied to intra- and inter-brain measures (2). Subsequently, the third phase (3) consisted in the calculation of a specific ConIndex as the ratio between inter-brain and intra-brain connectivity (Inter_con_/Intra_con_) that allowed to directly compare the two connectivity levels for single subject and subjects’ dyads. Finally, ANOVA was applied to ConIndex dependent measure (4).

## Results

### Behavioral data

Regarding questionnaire, two mixed-model ANOVAs were applied to cooperation self-perception and dyadic tuning scoring with the following factors: Block (pre vs. post) as repeated factor, Condition (Cond: order 1 vs order 2) and Role (Role: donor vs. receiver) as between factors.

Concerning dyadic tuning, ANOVA revealed a significant effect for Block (F[1,28] = 27.15; p < 0.0001; η2 = 0.79), with an increase in tuning perception after (M = 2.85; SD = 0.08) than before (M = 1.10; SD = 0.06) gift exchange. Concerning perceived cooperation, ANOVA revealed a significant effect for Block (F[1,29] = 34.89; p < 0.0001; η2 = 0.78), with an increase of cooperation perception after (M = 2.88; SD = 0.08) than before (M = 1.01; SD = 0.05) gift exchange.

For ACC measurement, ANOVA shown a significant effect for Cond (F[1,29] = 11.22; p < 0.001; η2 = 0.36), with an improvement of performance (higher percentages) for order 1 than order 2; Block (F[2,29] = 9.56; p < 0.001; η2 = 0.35) and Cond x Block (F[2,53] = 7.90; p < 0.001; η2 = 0.30). In particular, post-hoc comparison applied to interaction effect showed a higher ACC percentage for order 1 in block 2 more than block 1 (baseline) (F[1,29] = 11.77; p < 0.001; η2 = 0.34) and in block 3 more than block 1 (F[1,29] = 11.22; p < 0.001; η2 = 0.34).

**Figure 3:**
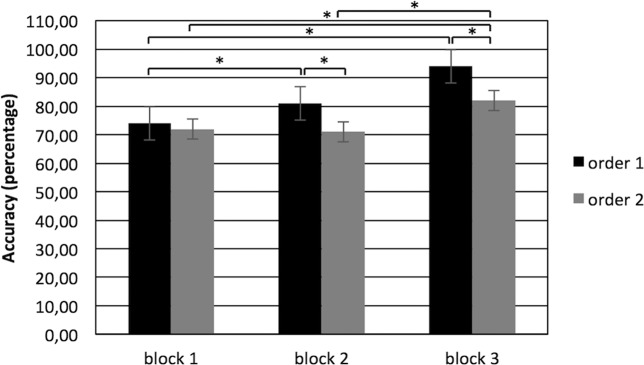
The figure shows an increase of performance accuracy in order 1 than order 2 in block 2 and in block 3, after gift exchange. Bars represent + −1SE. Stars mark statistically significant (p < 0.05) pairwise comparisons.

On the contrary, order 2 showed a higher ACC percentage in block 3 more than block 1 (F[1,29] = 11.70; p < 0.001; η2 = 0.35) and block 2 (F[1,29] = 10.09; p < 0.001; η2 = 0.33). Moreover, from the comparison between order 1 and 2, an increase of performance in order 1 than order 2 in block 2 (F[1,29] = 9.13; p < 0.001; η2 = 0.33) and in block 3 (F[1,29] = 10.34; p < 0.001; η2 = 0.35) was observed (Fig. 3).

Concerning RTs, ANOVA did not show any significant effect.

### fNIRS data

#### Intra-brain connectivity

A repeated measure ANOVAs was applied to O2Hb and deoxygenated hemoglobin (HHb) measures with Condition (Cond: order 1 vs order 2), Block (1 vs 2 vs 3) and ROI (4) as repeated factors.

**Figure 4:**
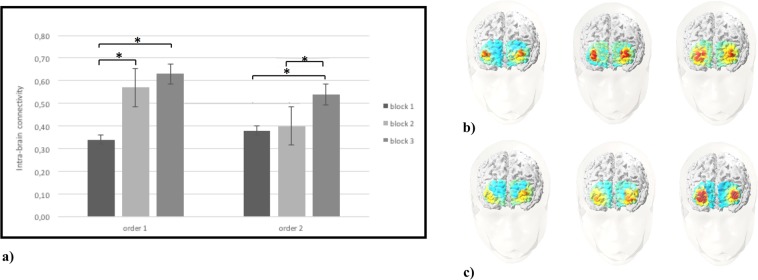
(**a**) The figure shows the values of O2Hb intra-brain connectivity in DLPFC area for order 1 and 2 during the execution of the three blocks of the task. Bars represent + −1SE. Stars mark statistically significant (p < 0.05) pairwise comparisons. (**b**) O2Hb intra-brain connectivity representation, from left to right, in order 1 for block 1, 2 and 3. The red area represents the increase of O2Hb intra-brain connectivity in DLPFC area. (**c**) O2Hb intra-brain connectivity representation, from left to right, in order 2 for block 1, 2 and 3. The red area represents the increase of O2Hb intra-brain connectivity in DLPFC area.

As shown by ANOVA applied to O2Hb data, Cond effect was significant (F[1,29] = 9.50, p < 0.01, η2 = 0.33), with a general increased intra-brain connectivity for order 1 more than order 2. In addition Cond x Block x ROI interaction effect was significant (F[6,72] = 7.90, p < 0.01, η2 = 0.29). In particular, as shown by post-hoc comparisons, there was an increase of intra-brain connectivity in DLPFC area for order 1 in block 2 and 3 than block 1 (respectively F[1,29] = 8.45, p < 0.01, η2 = 0.30; F[1,27] = 6.88, p < 0.01, η2 = 0.26) (Fig. 4a,b). Similarly, in order 2 block 3 showed an increase of DLPFC activity than block 1 and block 2 (respectively F[1,29] = 7.11, p < 0.05, η2 = 0.29; F[1,27] = 7.56, p < 0.01, η2 = 0.29) (Fig. 4a,c).

No significant effect was found for HHb data.

#### Inter-brain connectivity

**Figure 5:**
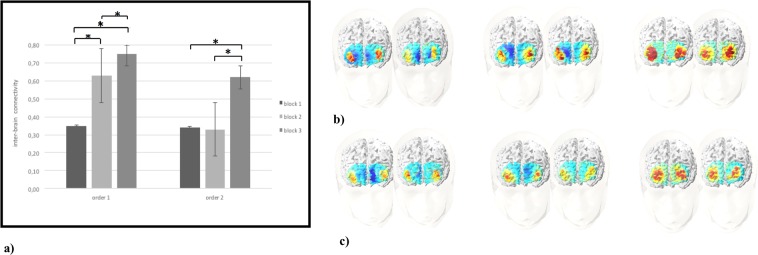
(**a**) The figure shows the values of O2Hb inter-brain connectivity in DLPFC area for order 1 and 2 during the execution of the three blocks of the task. Bars represent + −1SE. Stars mark statistically significant (p < 0.05) pairwise comparisons. (**b**) O2Hb inter-brain connectivity representation, from left to right, for donor and receiver during the execution of block 1, 2 and 3 in order 1. The red area represents the increase of O2Hb inter-brain connectivity in DLPFC area. (**c**) O2Hb inter-brain connectivity representation, from left to right, for donor and receiver during the execution of block 1, 2 and 3 in order 2. The red area represents the increase of O2Hb inter-brain connectivity in DLPFC area.

ANOVA applied to O2Hb data, showed a significant effect for Cond (F[1,29] = 9.77, p < 0.01, η2 = 0.33), with a general increased inter-brain connectivity for order 1 more than order 2. In addition Cond x Block x ROI interaction effect was significant (F[6,72] = 7.45, p < 0.01, η2 = 0.29). In particular, as revealed by post-hoc comparisons, an increase of inter-brain connectivity was observed in DLPFC area for order 1 in block 2 and 3 than block 1 (respectively F[1,29] = 8.98, p < 0.01, η2 = 0.30; F[1,27] = 7.67, p < 0.01, η2 = 0.26) (Fig. 5a,b). Moreover, in order 1 block 2 differed from block 3, with higher DLPFC connectivity in block 3 (F[1,29] = 8.76, p < 0.01, η2 = 0.32). Similarly, in order 2 block 3 showed increased DLPFC connectivity than block 1 and block 2 (respectively F[1,29] = 7.09, p < 0.05, η2 = 0.30; F[1,27] = 7.59, p < 0.01, η2 = 0.31) (Fig. 5a,c).

No significant effect was found for HHb data.

#### ConIndex

**Figure 6:**
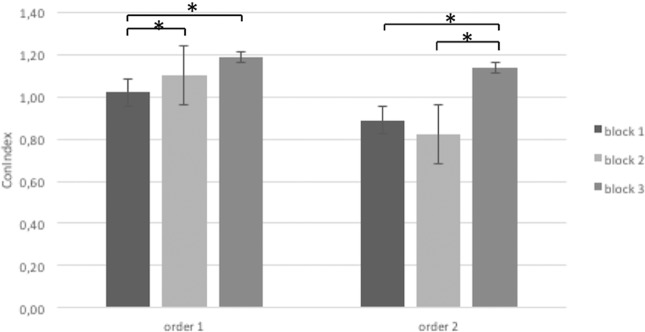
The figure shows ConIndex values for block 1, 2 and 3 in order 1 and order 2. Bars represent + −1SE. Stars mark statistically significant (p < 0.05) pairwise comparisons.

The ANOVA applied to ConIndex showed significant Cond × Block × ROI interaction effect (F[6,72] = 7.90, p < 0.01, η2 = 0.29). Specifically, post-hoc comparisons revealed an increased ConIndex (higher inter-brain connectivity than intra-brain connectivity) in DLPFC for order 1 in block 2 and 3 more than block 1 (respectively F[1,29] = 8.76, p < 0.01, η2 = 0.35; F[1,27] = 9.13, p < 0.01, η2 = 0.37). In addition, in order 2 an increased ConIndex in DLPFC was observed in block 3 more than block 1 and block 2 (respectively F[1,29] = 8.12, p < 0.01, η2 = 0.35; F[1,29] = 7.89, p < 0.01, η2 = 0.32) (Fig. 6).

## Discussion

The present research aimed to investigate if and how gift exchange could influence behavioral performance as well as neural activity through the increase of intra-brain and inter-brain connectivity between individuals involved in the social exchange based on prosocial condition of selfless gift.

Moreover, the comparison between intra-brain and inter-brain connectivity (ConIndex) allowed the investigation of the degree of inter-brain (inter-subject) compared to intra-brain (single subject) connectivity within specific cerebral areas. It was hypothesized to observe an increase of cooperation and a cognitive performance improvement following the gift exchange, with significant DLPFC increased intra-brain and inter-brain connectivity after gift exchange.

According to the first hypothesis, the results of the present study showed an increase of individuals’ behavioral responses, in terms of dyadic tuning, degree of cooperation self-perception, and cognitive performance following the gift exchange. We may suggest that the increase of prosocial and cooperative behavior experienced after gift exchange provide an increase of individuals’ attunement and interpersonal coordination, influencing behavioral performance.

Indeed, as demonstrated by previous studies^35,64,65^, the presence of a greater interpersonal link and cohesion lead to the implementation of a synergistic and cooperative behavior increasing the adoption of common strategies and perceived effectiveness that influence individuals’ cognitive level, improving also the performance level^68^.

Furthermore, the results of the present study have shown how, despite an increase of interpersonal cohesion and cognitive performance after the gift exchange, the effect appears to be strengthened when this moment takes place at the beginning of the task rather than in the middle. This phenomenon can be attributed to the implementation of prosocial behavior and reward mechanisms underlying cooperative bonds that stimulate the construction of social bonds^17^ and stronger reciprocity^20,21^ during the task execution, influencing social interaction from the beginning and being able to immediately induce a more significant modulations of cooperative and joined actions between the subjects^29,30,69^. Indeed we opted to compare two specific moments of the interpersonal exchange, an early moment and a late moment, in order to test the “priority effect” and “long lasting effect” of an early gift exchange. Indeed, our main aim was to test the effect induced by an early prosocial behavior (selfless gift) compared to a later one. The effect induced after the first block (order 1) or the second block (order 2) was a relevant test, and we expected to find a better performance, with a similar but higher improving result when the exchange was at the beginning.

This was due to the possibility to introduce an earlier relevant relational positive change with higher positive emotional and cognitive effect for the successive interpersonal dynamics across the time. Therefore, to opt for two clearly distinct times of gift exchange may evidence the significant effect of prosocial behavior on the relationships across the time.

The same trend was also found for neural responses. According to the second hypothesis, the results of the present study showed an increase of O2Hb intra and inter-brain connectivity in the DPLFC following the gift exchange. In particular, the increase of O2Hb intra-brain connectivity in DLPFC in both individuals (donors and receivers, without any differences based on the role) may be due to the fact that this brain area seems to be particularly involved in social interaction mechanisms, such as emotional attunement^32^, altruistic mind theory development and egoistic behavior suppression^40,70^. Furthermore, the involvement of DLPFC in social and relational processes is demonstrated by the regulation of empathic processes, social skills and social interaction processes^36,41,42,62,63^.

Similarly, concerning inter-brain connectivity, the results of the present study showed an increase of O2Hb inter-brain connectivity in DLPFC following gift exchange. This evidence underlines how this brain area turns out to be involved in interpersonal and social processes^40,70,71^. The increase of brain connectivity, indeed, can be seen as a marker of increased inter-neural synchronization, finalized to support the synergic activity of brain structures. Indeed, whereas connectivity refers to several different and interrelated aspects of brain organization^72^, functional connectivity, instead, is calculated between all elements of a system, regardless of whether these elements are connected by direct structural links. In addition, functional connectivity is highly time-dependent. In the present study, it is interesting to observe how the increase of inter-brain connectivity, besides the intra-brain one, in this specific cerebral area may be due to the involvement of mirroring mechanisms between donor and receiver due to an increase of cooperation provided by prosocial behavior consequent gift exchange. This evidence is supported by several studies that have observed that the development of common activities involves an alignment of individuals’ behavioral responses due to mechanisms of neural coupling^37,51^.

Finally, from the results of the ConIndex analysis, an increase of inter-brain connectivity compared to intra-brain emerged in the DLPFC area. This result confirms how the prosocial behavior experienced during gift exchange constitutes a joint action that increased the level of individuals’ attunement compared to the activation of a single brain per se. This result also underlines how the ConIndex analysis can provide useful information on the brain areas of greater connectivity, thus highlighting the contribution of the frontal region in mirroring and tuning mechanisms.

The present study, thus, highlights how the implementation of prosocial behavior, such as the gift exchange, strengthens interpersonal relationships by increasing cooperation and behavioral and neural coordination among individuals, underlying how the implementation of joint actions increases the mechanisms of intra and inter-cerebral connectivity. Furthermore, the use of the hyperscanning paradigm allowed us to obtain information on the mechanisms underlying the social and interpersonal relationships and on individuals’ levels of neural attunement. Despite the innovativeness of the study, we can highlight some weaknesses that could be implemented in future studies, such as a more targeted investigation on the role of the agents involved in the exchange (donor and receiver) and any differences related to participants’ gender. Furthermore, the integration of other neuroscientific tools and techniques could contribute to investigating the neurophysiological correlates underlying different emotional states and mechanisms characterizing social interactions. Finally, to better generalize the present results an ample sample size could be suggested for successive investigations. At present, power analysis supported the results as a pilot study, in absence of population as a reference for the sample size. Future research could consider gender effect with mixed couple male-female or only male, to investigate possible differences based on individuals’ gender.

Furthermore, in future research the strength of empirical observations and related interpretations would improve from replications with larger samples.

## Data Availability

The datasets generated during and/or analysed during the current study are available from the corresponding author on reasonable request.
